# A Neural Network-Based Gait Phase Classification Method Using Sensors Equipped on Lower Limb Exoskeleton Robots

**DOI:** 10.3390/s151127738

**Published:** 2015-10-30

**Authors:** Jun-Young Jung, Wonho Heo, Hyundae Yang, Hyunsub Park

**Affiliations:** 1 Robot Group, Korea Institute of Industrial Technology, 143 Hanggaul-ro, Sanrok-gu, Ansan-si, Gyeonggi-do 15588, Korea; E-Mails: eiever@kitech.re.kr (W.H.); hd0301@kitech.re.kr (H.Y.); hsubpark@kitech.re.kr (H.P.); 2 School of Electrical and Electronic Engineering, Yonsei University, 50 Yonsei-ro, Seodaemun-gu, Seoul 03722, Korea; 3 School of Intelligent Robots, University of Science and Technology, 217 Gajeong-ro, Yuseong-gu, Daejeon 34113, Korea; 4 System Industry PD Group, Korea Evaluation Institute of Industrial Technology, 32 Cheomdan-ro 8-gil, Dong-gu, Daegu 41069, Korea

**Keywords:** exoskeleton robots, gait phase classification, neural network, MLP, NARX

## Abstract

An exact classification of different gait phases is essential to enable the control of exoskeleton robots and detect the intentions of users. We propose a gait phase classification method based on neural networks using sensor signals from lower limb exoskeleton robots. In such robots, foot sensors with force sensing registers are commonly used to classify gait phases. We describe classifiers that use the orientation of each lower limb segment and the angular velocities of the joints to output the current gait phase. Experiments to obtain the input signals and desired outputs for the learning and validation process are conducted, and two neural network methods (a multilayer perceptron and nonlinear autoregressive with external inputs (NARX)) are used to develop an optimal classifier. Offline and online evaluations using four criteria are used to compare the performance of the classifiers. The proposed NARX-based method exhibits sufficiently good performance to replace foot sensors as a means of classifying gait phases.

## 1. Introduction

Walking involves periodic behavior in both legs, with one moving forward as the other bears the weight of the body, thus moving the whole body from one place to another [1]. Such motion is a common and essential behavior in humans. Although walking is not difficult for most people, babies, the elderly, and disabled may find it difficult to perform basic walking motions [2]. Babies typically require 11–15 months to learn to walk, whereas older people can encounter several problems related to muscle weakness, balance problems, and age-related diseases that prevent normal walking. Disabilities of the lower body or related body parts can also make it hard to walk. To understand the reasons behind different levels of walking difficulty, and to conceive methods to solve these problems, a walking analysis can be conducted.

Gait phase classification is a general method for analyzing walking. Walking is the periodic behavior of one stride, measured from one leg’s heel strike (ground contact) to the same leg’s next heel strike. One stride can be further divided into two phases, the stance in which one leg bears the weight of the body and the swing whereby the leg moves forward to propel the body [1]. Each phase can be spilt into four sub-phases for the stance and three sub-phases for the swing. The swing and stance phases have physically distinguishable cut-off points. The swing starts when one foot leaves the ground (Toe-Off) and ends when that foot next contacts the ground (Heel-Strike); the stance occurs between the Heel-Strike and the Toe-Off. However, there is currently no clear classification of the associated sub-phases. In this paper, we focus on the classification of two walking phases.

There are many applications of gait phase classification information. Based on how and where the information would be used, there are two main areas of application. First, as mentioned above, gait phase information can be used to evaluate and determine the walking pattern of humans. Traditionally, such information is used as quantitative data alongside other spatio-temporal parameters (e.g., stride width, walking speed, cadence, and walking symmetry) to diagnose and prescribe pathological gaits and evaluate walking after rehabilitation [1,2]. Second, gait phase classification results are used in several walking-aid devices. Recent advances in technology have seen the introduction of devices such as Functional Electronic Stimulation (FES) [3,4,5,6,7,8,9,10], lower limb exoskeleton robots [11,12,13,14,15,16,17], and smart, intelligent, or powered prostheses [18,19,20,21,22] to help people with walking disabilities.

Gait phase information is especially important for walking-aid devices. FES devices use this information to enable patients with spinal cord injuries to walk via the direct electronic stimulation of lower limb muscles. Lower limb exoskeleton robots are used to help people with walking disabilities and to enhance the performance of healthy subjects. When the robots walk with the user, the control model changes according to information about whether one or both feet are in contact with the ground. Thus, gait phase information is an essential control input. Similarly, in smart lower limb prosthetics, the damping coefficients in artificial knee joints must be changed to simulate those of the human so as to reduce energy consumption and allow lower-limb amputees to walk stably. Gait phase information is a vital component in this scenario.

To be of use in the above cases, gait phase information must be correctly classified. It is generally known that a force plate is the most effective means of correctly distinguishing between the two phases of walking. However, force plates have several non-practical features. For example, they are very expensive sensors. Measurements can only be taken at the place where the force plate is installed, and the gait phase is only detectable when a person stamps on the force plate while walking. For gait classification when using walking-aid devices, another important consideration is real-time classification. Although not essential for diagnosis, controlling walking-aid devices requires gait phase classification information to be acquired in real time. Thus, for walking-aid devices, more practical methods are needed.

The problem of gait classification can be generalized to that of determining what sensors should be used to acquire walking data and how the gait phase can then be classified. In this scenario, the most important factor is to select the most reliable and suitable sensors. Recent advances in wearable sensor technologies [23] have seen the development of various wearable sensor-based gait classification methods, enabling gait phases to be classified in an autonomous manner. These technologies include electromyogram sensors [19], which measure the electrical activity of muscles, Force Sensing Registers (FSRs) [4,8,22,23], goniometers [4], accelerometers [9,24], gyroscopes [8,25,26], and Inertial Measurement Units (IMUs) [20,22], which measure the force or displacement of movements. Several machine learning-based autonomous methods have been used to classify the gait phase using the signals from these sensors. For instance, support vector machines, artificial neural networks [24], hidden Markov models [19,25,26], and fuzzy models [27] have been employed, as have simple heuristic threshold methods [8,22].

Wearable sensor-based approaches have a number of advantages, e.g., they are not limited to a specific environment, can record any number of measureable steps, and are typically low cost*.* They also have some disadvantages when used with walking-aid devices, e.g., their classification success rate is lower than that of a force plate, their location on the body may clash with that of the walking-aid device, and accelerometers and gyroscopes must be strapped tightly to the body to acquire reliable data. In addition, multiple wearable sensors require a wired connection to the main controllers, as wireless connections have a limited bandwidth. This can be a significant burden for the wearers. Consequently, we believe that wearable sensors are not sufficiently mature to be used with exoskeleton robots and powered prostheses, but are well-suited to controlling FES.

Assistive devices such as exoskeleton robots and powered prostheses typically use embedded sensors to control themselves and detect the user’s intentions. These devices generally have encoders or potentiometers on their joints, IMUs, and foot sensors. Because foot contact is directly used to classify the gait phase, foot sensor-based approaches are commonly employed with exoskeleton robots and powered prostheses. However, it is difficult to make a foot sensor that is reliable and robust to different foot sizes and ground conditions. Furthermore, several exoskeleton robot structures cannot easily be fitted with foot sensors [28].

In this paper, we propose a neural network-based gait phase classification method that takes its input from non-foot sensor signals provided by exoskeleton robots. The purpose of the proposed method is to replace the general gait classification methods that use foot sensors. The orientation and angular velocities of lower limb segments are measured and estimated by the sensors, and the data are fed into neural network-based gait phase classifiers. We use two neural networks to determine the best model for classifying gait phases. Ten able-bodied subjects took part in an experimental study. To acquire training and offline validation data, seven people walked while wearing an exoskeleton robot. The developed classifiers were then evaluated in an online manner while the other three people used the exoskeleton robot. Our results show that the proposed method has the potential to replace foot sensor-based classifiers.

## 2. Methods and Materials

### 2.1. ROBIN-H1 Exoskeleton Robot and Its Sensor Configuration

In this section, we briefly introduce ROBIN-H1, a lower limb exoskeleton robot. The ROBIN-H1 system is used to acquire the walking data used to develop our gait phase classifier, and to verify the classifiers in an online manner. ROBIN-H1 was developed as a walking rehabilitation service for stroke patients. The specification of ROBIN-H1 is presented in Table 1.

**Table 1:** Specification of ROBIN-H1.

Feature	Specification
Weight	11 kg
Actuator Module	BLDC Motor (Kollmorgen)
	GearHead (Harmonic Driver Gear)
	Motor Drive (Elmo Motor Controller)
Degrees of Freedom (DOF) (each leg)	Active: 2 DOFs (hip and knee)
	Passive: 1 DOF (ankle)
Range of Motion (ROM)	Hip: −30 to 110°
	Knee: 0 to 110°
Equipped Sensors	2 IMUs
	4 absolute encoders
	4 incremental encoders
	2 foot sensors

**Figure 1:**
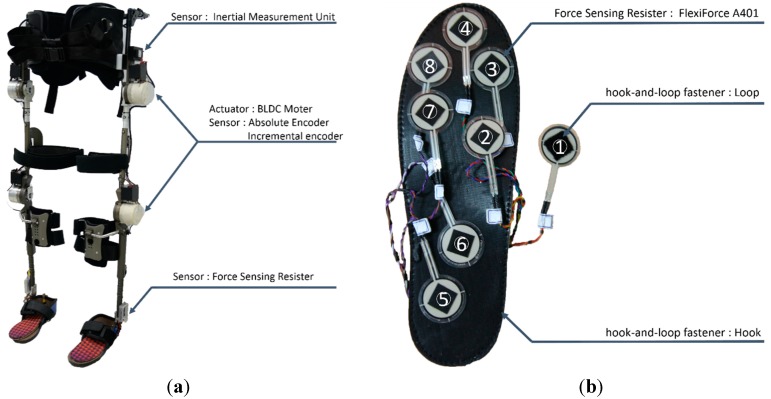
The appearance and sensor configurations of ROBIN-H1. (**a**) ROBIN-H1 lower limb exoskeleton robot and its hardware configuration; (**b**) Insole-type foot sensor configuration. Eight FSRs are inserted between the upper and lower insoles. The placement of each FSR is determined and adjusted according to the foot size and shape of the wearer before using the robot.

ROBIN-H1 is equipped with several sensors to detect walking intent and control itself (Figure 1). Absolute and incremental encoders are attached to the four active joints (both hips and knees) that move the legs in the sagittal plane. These measure the relative angle between the two lower limb segments. Both the left and right trunk segments have IMUs to measure the 3-axis angular velocities and accelerations of the body. These signals are fed to a trunk orientation estimator [29] to calculate the roll and pitch orientations of the frame. After determining the orientation of the trunk segments, the robot infers the pitch orientation of each lower limb segment. Each foot plate has insole-type foot sensors containing eight FSRs to detect whether the foot is in contact with the ground. We used the FlexiForce A401 FSRs, made by Tekscan (South Boston, MA, USA). Their known force sensing range is 0–3175 kg, but we use this to measure forces from 0–11 kg (110 N) by adjusting the drive voltage and resistance values of the drive circuit. The FSR outputs a voltage value that is transformed to kilograms using a mapping function provided by the manufacturer. The FSRs are easily movable inside the sensor to adapt to different foot sizes and pressure points. Three FSRs were placed at the heel area. Another four FSRs were placed at the metatarsal area. The final FSR was assigned to the toe area according to the foot size of the wearer.

The robot has a sensing mode in which various walking parameters of the human using the robot can be measured. In this mode, none of the active joints force the user’s body, but instead compensate for the weight of the exoskeleton and the friction in each active joint. Thus, the user can walk without feeling the additional weight and friction if he/she does not walk too fast. For compensation, we used two models. Equation (1) describes the motion model of ROBIN-H1:

$$\tau =B\left(q\right)\ddot{q}+C\left(q,\dot{q}\right)\dot{q}+\tau_{f}+g\left(q\right)$$

(1) $$⋍\;\tau_{f}+g\left(q\right)$$

where τ is a vector of the desired torque of each joint; B(q) is an inertia matrix term; q is a vector of the current angle of each joint; $C\left(q,\dot{q}\right)$ is the centrifugal and Coriolis forces matrix term; $\tau_{f}$ is the friction term of each joint; and g(q) is the gravity term. To offset the effect of gravity, a variety of information is necessary: the mass and position of the center of mass of each link (segment), gait phase information, and the pose of each link. The mass and position of the center of mass was calculated by a 3D-CAD program according to the segment design. Gait phase information was detected by a foot sensor-based gait phase classification method that is described in detail in the next paragraph. The pose of each link was obtained from forward kinematics using the sensor data, such as the IMU and absolute encoders. For the friction compensation of each joint, the following simple friction model was used [30]:

(2) $$\tau_{f}=f_{c}\text{sgn}\left(\dot{q}\right)+f_{v}\dot{q}$$

where $f_{\text{c}}$ is the Coulomb friction coefficient and $f_{v}$ is the viscous friction coefficient. These parameters are determined from the relation between the input torque in a motor driver and the angular velocity yielded at the torque. The Coulomb friction parameter is the torque, and the viscous friction parameter is the slope of the relation between the torque and the angular velocity. The slope is calculated by the least-squares estimation method.

Foot Sensor-based Gait Classification: As mentioned above, to operate in this mode, gait phase information is required for each leg. We use foot sensor signals to classify the gait phase in the experimental stages. To classify the gait phase, the FSR outputs are first aggregated. If the total is above some threshold, the robot is considered to be in the stance phase. The threshold is determined heuristically from several walking trials. The foot sensor signal-based gait phase classification logic is executed at 10 ms intervals.

The main controller of the robot is a personal computer. The controller operating system is Microsoft Windows 7, and the robot control software is written in C++. Communication between the main controller, motor controllers, and sensor boards is conducted via a Controller Area Network (CAN). Measured values are filtered by a second-order Butterworth filter at suitable frequencies. The sensed values and states of the robot are recorded at 66.7 Hz and saved to file. To synchronize with other devices such as force plates, the robot has a synchronization device based on two FSRs.

### 2.2. Data Acquisition

To develop a gait phase classifier and verify its performance offline, we first collected walking data. The walking data were acquired from able-bodied volunteers walking across three force plates while wearing ROBIN-H1. When a person walks while wearing an exoskeleton robot, it is known that the kinematics and kinetics of walking are affected by the weight and limited operation range of the robot [31]. Thus, if we wish to develop a gait phase classifier for a robot, we should use data acquired from walking while wearing the robot. To acquire walking data, ROBIN-H1 was operated in sensing mode to compensate for its weight and the friction of each joint.

**Figure 2:**
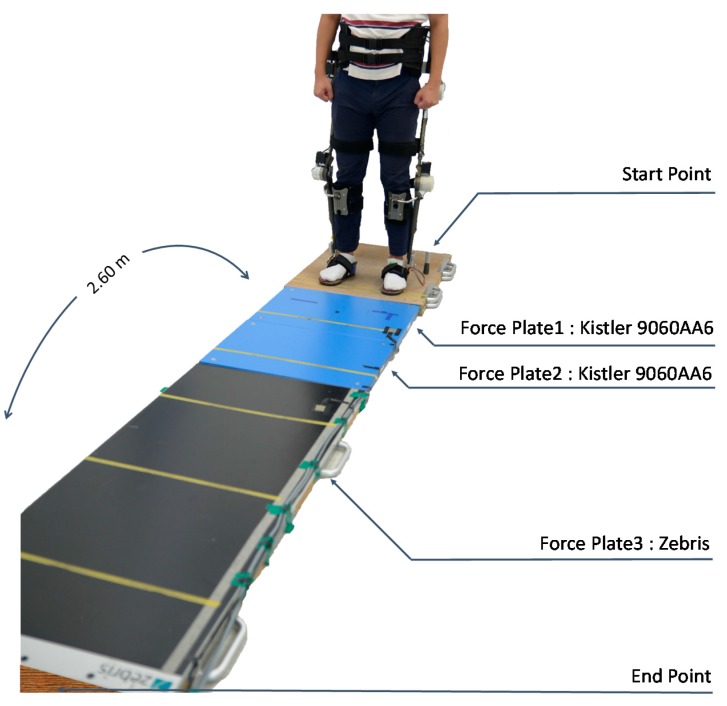
Configuration of the environment for the walking experiments. Total walking length from start point to end point was 2.60 m. Three force plates were used to acquire ground reaction forces while walking. The subjects start walking at the start point and end walking at the end point while maintaining a standing posture.

Seven healthy people who have not suffered any lower limb related disabilities took part in the data acquisition experiment. The experiment was approved by the Public Institutional Review Board of Korea. Before the experiment, we informed the participants about the nature of the experiment, and obtained written consent from each subject. Figure 2 shows the experimental environment.

The experiments were configured as follows:

(1) Each subject wears the ROBIN-H1 device and stands at the start position.
(2) A synchronization process is conducted between the robot and the force plates.
(3) Upon the start signal, the subject starts to walk by moving their right leg forward.
(4) The subject selects a convenient walking speed and step size.
(5) The subject takes four or five steps, and finally stands still after moving their left leg.
(6) Each subject repeats six trials.

It is possible to divide the data acquired from the above procedure into three categories. The first category is the robot data measured by the sensors on the robot and estimated by the robot’s algorithms. The remaining categories consist of force plate data from the Kistler 9060AA6 plates and the Zebris plate. Although the Kistler and Zebris force plates have different data acquisition software and formats, both types of force plate record their measurement values every 10 ms. Because the acquired data are different, some method of synchronization is needed. ROBIN-H1 has a synchronization device that uses two FSRs. These FSRs are attached to each type of force plate before the experiments. In the synchronization process, we briefly press down strongly on the FSR and the force plate. ROBIN-H1 and both types of force plate record this signal and its occurrence time.

### 2.3. Pre-Processing and Feature Selection

We first pre-processed the acquired data to transform them into feasible information for machine learning. In this study, we constructed training and validation datasets, and normalized the raw data in the pre-processing step. The three heterogeneous data points (one from each category) were combined into a single point in a time series data using a synchronizing signal. By synchronizing the start points of the three data streams, a synchronized time series was generated using the “synchronize” function of Matlab 2015a. To remove data recorded while standing still (at the start and end points), we cut the time series into several shorter series, and gathered the data from a single right-leg stride into one dataset. The reference points for cutting the data were obtained from the force plate data. In this manner, we obtained time series data set for 78 strides. This was divided into a training dataset T and a validation dataset *V*:

(3) k=m+n

(4) $$T=\left\{\left(x_{1},y_{1}\right),\left(x_{2},y_{2}\right),\cdots \left(x_{i},y_{i}\right)\right\}\;,\;i=1,\;2,\;3\;\cdots m$$

(5) $$V=\left\{\left(x_{1},y_{1}\right),\left(x_{2},y_{2}\right),\cdots \left(x_{i},y_{i}\right)\right\}\;,\;i=1,\;2,\;3\;\cdots n$$

where *k* is the total number of frames in the dataset; *m* is the number of frames in the training set and *n* is the number of frames in the validation set.

The machine learning method employed in this paper is a sort of supervised learning technique in which the training and validation sets are composed of pairs of input data x and desired output y. The inputs x used for the training and validation of our gait phase classifier were selected from the robot data. The robot data contain many parameters to describe the movement of the lower limbs. Among this data, we selected the pitch orientations of the right upper leg $x_{ul}$, thigh) and right lower leg ($x_{ll}$, shank) and their angular velocities ($\dot{x_{ul}},\;\dot{\;x_{ll}}$) to form the input vector $x_{\text{k}}$:

(6) $$x_{\text{k}}=\left[\begin{array}{c} x_{ul}\\ \begin{array}{c} x_{ll}\\ \dot{x_{ul}}\\ \dot{x_{ll}} \end{array} \end{array}\right]$$

Note that the input vector x uses orientation information from each leg, rather than the angles of each joint. An over-ground walking exoskeleton robot is not fixed with respect to the environment, but is fixed to the body of the user. Hence, angular information is affected by the inclination of the user’s trunk segments. The orientation of each lower limb segment is calculated from the estimated trunk segment orientation. The robot system estimates the trunk orientation in real time using IMU. The angular velocities of the segments are calculated by differentiating the joint angles at 5 ms intervals.

The desired output *y* in the training and validation datasets was generated by the force plate data. As described previously, the best information for classifying the swing and stance phases is the output from force plates inserted in the ground. If the force plate measured a value of more than 5 kg (in the F_z_ axis), we considered the state to be a stance and set *y* = 1; otherwise, we considered the phase to be the swing, and set *y* = 0:

(7) $$y=\{\begin{array}{c} 1,\;stance\;or\\ 0,\;swing\; \end{array}$$

After forming the training and validation datasets, we normalized the data. Neural networks can be adversely affected by the elements of the input vectors having a wide range of values. To overcome this problem, it is common to apply some form of normalization to restrict the input vector elements to the range [−1, 1], *i.e.*,

(8) $$x_{i}≔\left(y_{max}-y_{min}\right)\times (x_{i}-x_{min})/\left(x_{max}-x_{min}\right)$$

where $x_{i}$ is the *i*th element of input vector x, and $y_{max},\;y_{min}$ denote the maximum and minimum values of the elements in a normalized vector. In this case, we set the maximum and minimum to 1 and −1, respectively. $x_{min}$ is the minimum value of the *i*th element in the whole input vector set, and $x_{max}$ is the maximum value of the *i*th element in the input vector set.

### 2.4. Development of Gait Phase Classifiers

We developed gait phase classifiers using the training dataset. To classify the gait phases, we used two neural network methods, a multilayer perceptron (MLP) and nonlinear autoregressive with external (exogenous) inputs (NARX). MLP networks in which cross-entropy activation functions are used in the output layer are commonly employed for pattern recognition and classification. Walking can be considered as a temporal process, and the current state is dependent on previous states. Thus, walking can be regarded as a dynamical system. The best way to mimic a dynamical system is to use a dynamic recurrent neural network such as NARX. The training algorithm for these networks is generally some variant of back-propagation.

Because the gait phase classifiers proposed in this paper are integrated into the ROBIN-H1 control software to control the robot and detect the user’s intentions, high classification accuracy is essential. There are several design factors that must be considered to optimize the performance of a neural network used as a function-mimicking tool [32,33]. It is known that the capacity of a neural network to model complex behavior increases with the number of hidden layers. However, too many hidden layers can lead to problems such as overfitting and excessive training times. The number of nodes in the hidden layers must also be considered. Too few nodes in the hidden layers can limit the network’s ability to reproduce the target function’s behavior, whereas too many can result in overfitting. In addition, a suitable number of learning iterations is also important. For the NARX network, the input time delay also influences the performance [34].

**Figure 3:**
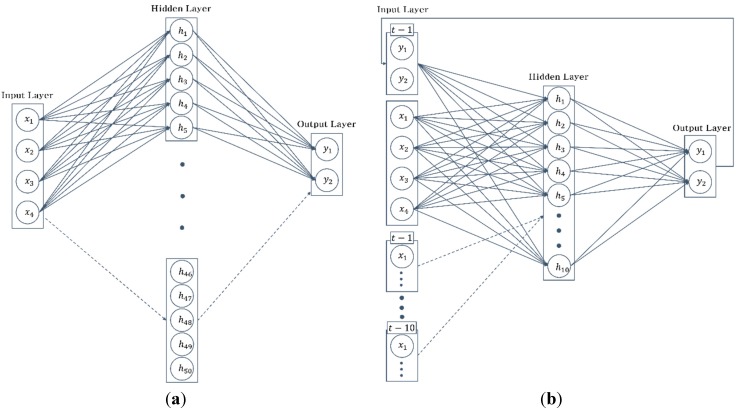
Growing network model (structure) of neural networks. (**a**) MLP-based network, with an increasing number of nodes in the hidden layer. The number of nodes increases from 5 to 50 nodes; (**b**) NARX-based network with an increasing number of nodes in the input layer as the input time delay increases from 1 to 10. Each input time delay has four nodes.

In this study, we applied a growing network model (structure or architecture) strategy to develop the best classifier for ROBIN-H1. The growing network model strategy only deals with the number of nodes in the hidden layer of the MLP and the number of nodes (the number of input time delays) in the input layer of NARX. Among the design factors, the number of hidden layers is not considered in this paper, because the input vector in our problem is only four-dimensional, and increased numbers of hidden layers are commonly necessary when the dimension of the input vector or the number of nodes in the input layer are large [35]. The design factor of the number of training iterations has also been neglected. This factor is autonomously determined to avoid overfitting the neural network with Matlab 2015a, which will be described in more detail in the next paragraph. The growing network model strategy is as follows. For the MLP network, we trained 10 networks by increasing the number of nodes in the hidden layer from 5 to 50. Other design factors of the MLP were kept fixed. Every MLP-based classifier had one hidden layer. In the NARX network, the number of hidden nodes was fixed, while the number of input neurons was varied. We trained 10 networks with an increasing number (1–10) input time delays. Consequently, the number of nodes in the input layer increased from 6 to 42. This is similar to a moving window method in which the window width must be determined. Figure 3 illustrates the growing network model strategy.

The neural network gait phase classifiers were trained using the Neural Network Toolbox in Matlab 2015a. The “patternnet” function of Matlab 2015a was used to generate MLP-based gait classifiers, and “narxnet” was used to make the NARX-based gait classifiers. To use the “patternnet” function for this classification problem, the output should have two elements: the desired output and the complementary value of the desired output. The weights of the neural network were initialized to random values in the range [−1, 1]. In some cases, training algorithms such as scaled conjugate gradient descent or Levenberg–Marquardt cannot find the global minimum because of the initial weights. To solve this local minima problem, we generated 30 networks for each model with different initial weights. The model with the best performance was then chosen to represent the network structure. The training algorithm was back-propagation based on conjugate gradient descent. The maximum number of learning epochs (iterations) was set to 1000. However, to avoid overfitting, the training process was terminated if the error between successive epochs increased by a factor of more than six.

## 3. Results and Discussion

### 3.1. Consideration of Evaluation Criterion

To compare the gait phase classifiers, we require some evaluation criterion. Generally, the Classification Success Rate (CSR) is used to validate a classifier. However, additional evaluation criteria are needed to consider certain characteristics of exoskeleton robots, such as the comfort and safety of the user, because the classifiers proposed in this paper are being used to control the robot. To develop an additional evaluation scale, we examined the types of error that appear in gait phase classifiers.

**Figure 4:**
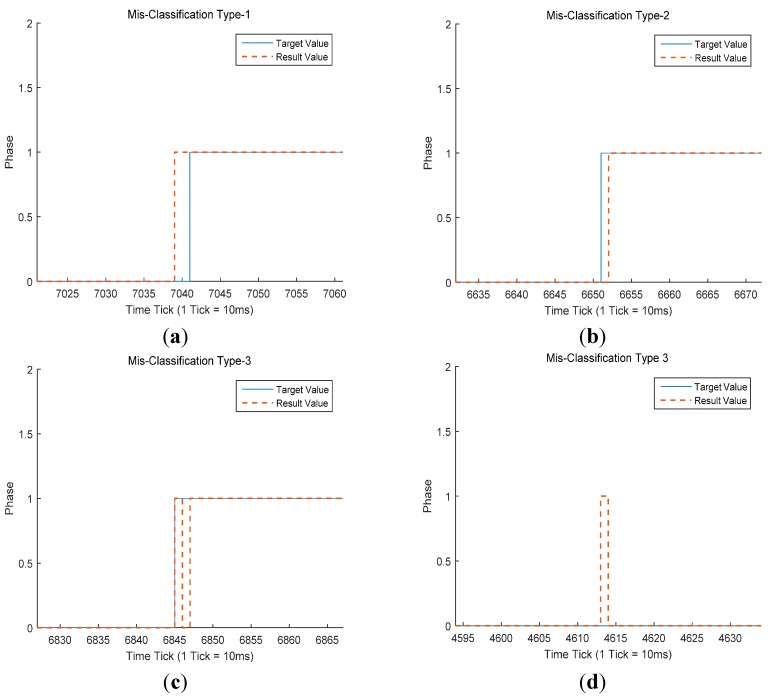
Misclassification types of a gait phase classifier. The blue line denotes target values acquired by the force plate. The orange dotted line denotes the classification result. (**a**) Early classification, misclassification type 1; (**b**) late classification, misclassification type 2; (**c**,**d**) unstable regions, misclassification type 3.

The errors can be categorized as follows (Figure 4):

(1) The classifier considers a gait phase to have changed before the transition point in the real gait phase (Misclassification Type-1, Figure 4a).
(2) The classifier considers the gait phase to have changed after the transition point in the real gait phase (Misclassification Type-2, Figure 4b).
(3) The classifier considers the gait phase to have changed, but no transition has occurred in the real gait phase (Misclassification Type-3, Figure 4c,d).

In the case of applying a gait phase classifier to a robot, the extent to which each error type influences the user and the robot will be different. Thus, it is useful to impose a weight on each error type. However, when we calculate the CSR, we simply sum the number of errors and divide the sum by the number of whole frames. Misclassification types 1 and 2 do not affect the robot’s control logic, as long as the continuous error width is not too large (≥15). However, misclassification type 3, in which the classification output oscillates over a short time interval, is not suitable for robot control. For example, when the real gait of the user and the robot is in the stance phase, but the robot recognizes this as the swing phase and operates inappropriately, the user may feel uncomfortable, and could even fall down*.* Thus, all else being equal, a classifier with no unstable regions is regarded as the best classifier for exoskeleton robots. In this study, in addition to the CSR, we applied the following three evaluation criteria:

(1) Max Continuous Error Width: the maximum continuous error between the force plate results and the classification result.
(2) Mean and Standard Deviation (Std.) Continuous Error Width: the mean of the continuous error and its standard deviation.
(3) Number of unstable regions: the number of cases in which the classification results oscillate.

### 3.2. Offline Experimental Results

To compare the offline performance of the classifiers described in Section 2.4, we used the validation dataset described in Section 2.3. Validation dataset V consisted of 38 strides over 7221 frames. The classification results of each classifier are presented in Table 2 and Table 3.

**Table 2:** Offline classification results of walking phase classifiers with MLP networks.

Number of MLP Hidden Nodes	CSR (Error Rate)	Maximum Continuous Error Width (Frames)	Mean and Std. Continuous Error Width (Frames)	Number of Unstable Regions
5	97.70% (2.30%)	10	2.20 ± 1.78	3
10	97.73% (2.27%)	8	2.17 ± 1.56	4
15	97.71% (2.29%)	8	2.19 ± 1.62	8
20	97.73% (2.28%)	10	2.17 ± 1.63	4
25	97.76% (2.24%)	9	2.15 ± 1.65	3
30	97.85% (2.15%)	10	2.05 ± 1.55	4
35	97.76% (2.24%)	9	2.15 ± 1.66	4
40	97.78% (2.22%)	9	2.12 ± 1.61	3
45	97.74% (2.26%)	8	2.17 ± 1.56	5
50	97.71% (2.29%)	8	2.18 ± 1.40	6

**Table 3:** Offline classification results of walking phase classifiers with NARX networks.

Number of Input Time Delays	CSR (Error Rate)	Maximum Continuous Error Width (Frames)	Mean and Std. Continuous Error Width (Frames)	Number of Unstable Regions
1	97.95% (2.05%)	12	1.96 ± 1.79	0
2	97.70% (2.30%)	12	2.21 ± 1.76	0
3	97.78% (2.22%)	13	2.13 ± 1.81	0
4	97.74% (2.26%)	13	2.17 ± 1.83	0
5	97.71% (2.29%)	11	2.19 ± 1.76	0
6	97.73% (2.27%)	12	2.19 ± 1.78	0
7	97.53% (2.47%)	11	2.36 ± 1.86	0
8	97.60% (2.40%)	12	2.31 ± 1.91	0
9	97.50% (2.50%)	12	2.39 ± 1.93	0
10	97.38% (2.62%)	10	2.51 ± 2.01	0

**Figure 5:**
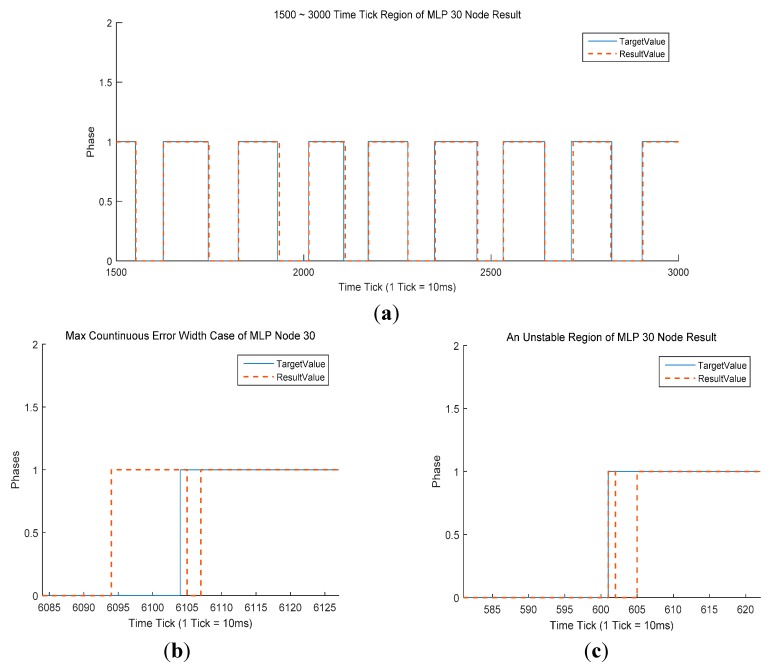
(**a**) Comparison of results and target value for the MPL with 30 nodes over frames 1500–3000. The blue line denotes the target value acquired by the force plates. The orange dotted line denotes the classification result of the MLP with a 30-node classifier, the best MLP-based classifier; (**b**) Max continuous error width for the MLP with 30 nodes; (**c**) An unstable region for the MLP with 30 nodes.

It is clear that the MLP classifiers produced excellent results (Table 2), with an average CSR above 97% (97.75%). The average maximum continuous error width was also good, at just 8.9 frames. The mean continuous error width was 2.16 frames, indicating that the difference in recognition time is less than 30 ms. This would enable the control of an exoskeleton robot. Although the MLP-based classifiers produced good results in three criteria, they performed poorly in terms of the number of unstable regions, with an average of 4.4. The best MLP classifier had 30 nodes in the hidden layer (Figure 5). This classifier gave the highest CSR (97.85%) and had only four unstable regions across the whole 38 strides. However, the maximum continuous error width was 10, the lowest result in the comparison. This indicates a recognition time difference of 100 ms. As shown in Figure 5b, this maximum continuous error occurred as the gait phase transitioned from swing to stance, that is, a heel strike recognition. In this case, an unstable region also occurred (Figure 5c).

**Figure 6:**
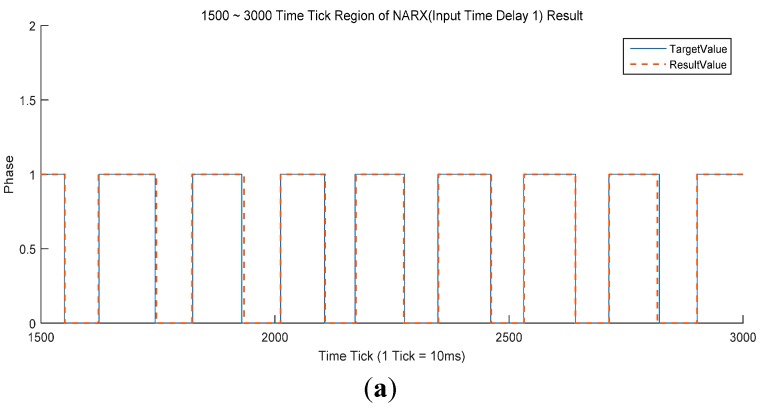

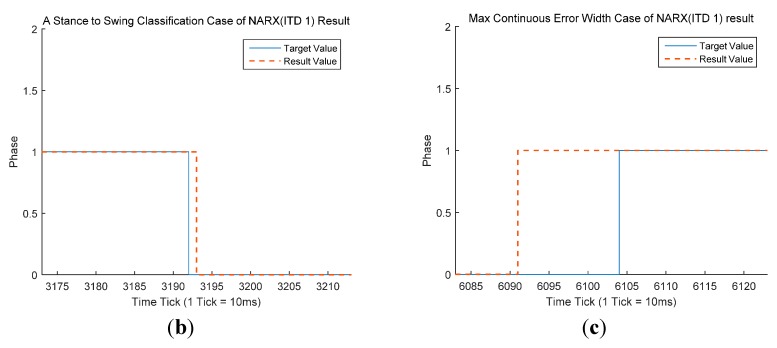
(**a**) Comparison of NARX results with an input time delay of 1 and the target values for frames 1500–3000. The blue line denotes the target value acquired by the force plates. The orange dotted line denotes the classification result of the NARX with ITD 1 classifier, the best NARX-based classifier; (**b**) General stance to swing classification for the NARX classifier in (**a**); (**c**) Max continuous error width for the NARX classifier in (**a**).

There does not appear to be any significant difference in CSR between the MLP-based and NARX-based (97.05%) classifiers (Table 3). The NARX-based classifiers gave a slightly larger maximum continuous error width and a slightly lower mean continuous error width than the MLP-based classifiers, but the differences were small. The most dramatic difference is in the number of unstable regions. In the NARX case, there are no unstable regions. This suggests that the NARX-based classifiers are more acceptable to exoskeleton robots than MLP-based classifiers. The best NARX-based classifier was that with a single input time delay (Figure 6). This classifier had a CSR of 97.95%, the best of all the MLP and NARX classifiers. In addition, this classifier had the lowest mean continuous error width and Std. However, the maximum continuous error width of 12 was somewhat large. The NARX-based classifiers also exhibited worse overall performance in the swing to stance case (Figure 6b,c).

### 3.3. Online Experimental Results

We implemented the gait phase classifiers on ROBIN-H1, and validated their online performance. It is generally known that the online performance of classifiers is worse than their offline performance. Hence, online validation can be considered as a practical means of identifying the performance of classifiers. For online validation, three subjects who did not attend the data acquisition experiments were asked to wear ROBIN-H1 and complete the same process of the previous experiments. The classifiers developed using Matlab 2015a were implemented in C++ in the robot control software by extracting weight parameters from the generated scripts. In the robot control software, the classification logic was operated periodically at 10 ms intervals. A comparison of the real-time output from the classifier and the force plate measurements for a total of 32 strides is presented in Table 4 and Table 5.

**Table 4:** Online classification results of walking phase classifiers with MLP networks.

Number of MLP Hidden Nodes	CSR (Error Rate)	Maximum Continuous Error Width (Frames)	Mean and Std. Continuous Error Width (Frames)	Number of Unstable Regions
5	91.46% (8.54%)	37	8.89 ± 8.18	3
10	90.38% (9.62%)	37	10.02 ± 9.22	12
15	91.08% (8.92%)	37	9.29 ± 8.73	4
20	90.85% (9.15%)	37	9.52 ± 8.91	7
25	88.35% (11.65%)	37	12.13 ± 11.20	15
30	91.51% (8.49%)	37	8.84 ± 8.62	1
35	90.51% (9.49%)	37	9.87 ± 9.06	9
40	91.14% (8.86%)	37	9.22 ± 8.68	6
45	91.44% (8.56%)	37	8.90 ± 8.22	3
50	90.82% (9.18%)	37	9.56 ± 8.67	4

**Table 5:** Online classification results of walking phase classifiers with NARX networks.

Number of Input Time Delays	CSR (Error Rate)	Maximum Continuous Error Width (Frames)	Mean and Std. Continuous Error Width (Frames)	Number of Unstable Regions
1	92.48% (7.52%)	28	7.82 ± 6.19	4
2	92.37% (7.63%)	31	7.94 ± 5.88	2
3	92.82% (7.18%)	34	7.48 ± 7.09	0
4	92.08% (7.92%)	34	8.24 ± 7.54	6
5	92.94% (7.06%)	28	7.35 ± 5.34	0
6	93.63% (6.37%)	27	6.63 ± 4.92	0
7	88.78% (11.22%)	33	11.68 ± 9.13	21
8	93.43% (6.57%)	34	6.84 ± 6.17	0
9	88.47% (11.53%)	41	12 ± 10.84	3
10	92.28% (7.72%)	28	8.03 ± 6.44	13

As expected, the MLP-based classifier exhibited worse overall performance than in the offline case. The average CSR (90.75%) produced by the online (Table 4) MLP networks was approximately 7% lower than in the offline case (Table 2). In addition, the other criteria were worse in this case. The best MLP-based classifier again had 30 nodes in the hidden layer.

Similarly, the NARX-based classifiers performed worse (Table 5) than in the offline validation (Table 3). Although both neural network-based classifiers failed to produce excellent results, the decrease in the range of performance of the NARX-based classifier is smaller than that of the MLPs. The NARX models produced an online CSR that averaged 91.93%, some 5.7% down on their offline performance. The maximum continuous error width and the mean and Std. of the continuous error also increased. The main advantage of the NARX-based classifiers in the offline experiment was the absence of unstable regions. However, in the online case, several NARX-based classifiers produced unstable regions.

The NARX-based classifiers gave better overall performance than the MLPs. Unlike the offline case, the best online NARX-based classifier had six input time delays.

Finally, Table 6 presents the classification results from an online foot sensor-based method. As described in Section 2.1, ROBIN-H1 has a series of foot sensors that can be used to classify the gait phase. This gait phase information was fed to the robot controller during the sensing mode.

### 3.4. Discussion

In this paper, two network-based classifiers have been evaluated in terms of their offline and online performance. It is clear that the overall performance of the classifiers was better in the offline case. The reasons for this are as follows.

First, the validation data were obtained from different subjects in each experiment. We doubt whether training data T can represent all walking patterns. When we develop a classifier using machine learning techniques, it is important to obtain representative data. However, even if walking pattern data for every person in the world were simply included in T, it would not be a representative set of walking data; instead, T should include more noise. Therefore, we require an appropriate method to acquire representative data. Additionally, walking while wearing an exoskeleton robot will influence different people in different ways, making it difficult to acquire representative walking patterns. Interestingly, every case of the maximum continuous error width occurred on only certain stride patterns in both the online and offline experiments. Comparing these patterns with the training set patterns may be linked to finding representative walking pattern data.

Second, there is a discontinuity in the training dataset T. In the pre-processing procedure described in Section 2.3, we cut the synchronized time series data into several pieces relating to individual strides to maximize the performance of the classifiers. However, when a person walks with the robot, there are no discontinuities in the gait pattern. We believe the discontinuities have more of an influence on the NARX-based classifiers. In the online case, the maximum continuous error width of the NARX-based classifiers was larger than that of the MLP-based classifiers. To overcome this problem, many force plates are needed to capture more continuous strides. In addition, a method that smoothly connects heterogeneous stride patterns is also needed. Because force plates are very expensive sensors and must be installed in a suitable environment, it is difficult to build an ideal sensing environment. The other sensors are not as precise as the force plates. Additionally, the connection between two individual stride patterns is not practical. The pattern of the connected parts may differ from that of a human walking pattern.

We believe that the classifiers proposed in this paper are sufficient for use as part of the control logic of exoskeleton robots, despite the fact that their online performance is worse than their offline results. The CSR remains at an acceptable level of above 90%, and the maximum continuous error width is above 35, indicating time differences of about 350 ms between the real and classified phases.

This may cause a problem with the robot, but it is unlikely to be critical, because the worst case is rare. The mean of the continuous error width is about 8, corresponding to an 80 ms time difference. This time difference is very short, and would be difficult for a human to recognize.

Finally, the best MLP-based classifier exhibited only one unstable region, whereas the best NARX-based classifier had no unstable regions. The performance of the proposed classifiers is better than that of foot sensors using FSRs (Table 6). As described in previous sections, this foot sensor-based gait phase classifier was operated in the sensing mode of ROBIN-H1 for training and offline validation data acquisition and online validation experiments. In addition, there are a number of disadvantages to using foot sensors, e.g., we have to measure the foot size of the user and modify the position of the FSRs to be below the maximum pressure points. The classifiers proposed in this paper use the sensors installed on exoskeleton robots to provide their input, and produce superior classification results to foot sensors. Unlike foot sensors, the proposed classifiers are robust to misclassification problems caused by the sliding or shifting of weight from one foot to the other.

**Table 6:** Online classification results of walking phase classifiers with foot sensors.

Sensor-Type	CSR (Error Rate)	Maximum Continuous Error Width (Frames)	Mean and Std. Continuous Error Width (Frames)	Number of Unstable Regions
Foot Sensors	91.44% (8.56%)	27	9.02 ± 5.21	1

To determine the optimal network structures of both neural network-based classifiers, we applied a growing network structure model method (in the case of MLP, the hidden node size was varied. In the case of NARX, the input time delay size was varied). Varying the network structure is a well-known strategy in the neural network literature, so we expected a dramatic difference in classification performance between the optimal structure and others. However, there were no significant differences between similar networks in the offline evaluation. In the online evaluation, there was a 3% difference in CSR for the MLP and a 5% difference in CSR for the NARX between the best and worst structures. Thus, no meaningful tendency was observed. We can carefully infer that this phenomenon is caused by the combination of two factors. First, the problem of gait phase classification using lower leg orientations and angular velocities seems a rather well-defined problem. The input vector and outputs have few dimensions. There are few stochastic and subtle properties in the decision boundaries to transform the input vectors to outputs. Second, we think that the mechanism that prevents overfitting in the Neural Network Tool Box of Matlab 2015a could cause this phenomenon. In the training process, the mechanism checks whether overfitting has occurred in every training epoch. If no overfitting has occurred, the mechanism continues training up to the pre-defined training iteration number limit. Conversely, if overfitting has occurred six times in a row, the mechanism stops training and rolls back the weights of the network to the values prior to the occurrence of overfitting. This automatic process to determine the number of training iterations means that different structures will exhibit similar performance if the network structures are sufficient to simulate the problem. Although the growing network structure model strategy fails to identify much difference in performance between the best and worst structures, it provides an answer as to how the best network structures could be determined.

Finally, this paper shows that, in the case of modeling a dynamical system, the NARX network is better than the MLP network and that walking is a dynamical system. However, the time delay inputs and recurrent outputs in the input layer of NARX that make the NARX network good at modeling dynamical systems also cause the network to be easily affected by discontinuities in the training dataset and sensor noise in online operation. This means that it is difficult to make a high-performance NARX network-based classifiers. For this reason, further studies on constructing the training dataset, eradicating noise, and configuring the initial condition of the NARX network are needed.

## 4. Conclusions

This paper has proposed two neural network-based gait phase classifiers. Because the proposed classifiers will form part of the control logic of an exoskeleton robot, we acquired data from able-bodied people wearing exoskeleton robots to train the two networks. To find the optimal network structure, we trained the networks according to a growing network model strategy. We considered four evaluation criteria and assessed the offline and online performance of the classifiers. The proposed classifiers produced excellent offline results (average CSR: 97%), but their online performance was not so strong. The proposed classifiers outperformed foot sensor signal-based gait phase classification, despite their slightly lower online performance. The best NARX-based classifier did not produce any unstable regions, and exhibited good overall performance. Thus, NARX is an efficient and effective means of modeling the behavior of a dynamic system such as walking.

Although the proposed gait classifiers appear to be sufficiently accurate and robust to replace foot sensors in exoskeleton robots, some further improvements could be made. For example, we intend to determine a suitable method of acquiring representative walking pattern data. Secondly, we will apply a more advanced classification method, such as an auto-encoder. Finally, we will validate the proposed classifiers against data from stroke patients.
